# Pro-Inflammatory Biomarkers and Progression of Atherosclerosis in Patients with Myocardial Infarction with Non-Obstructive Coronary Artery Disease: 1-Year Follow-Up

**DOI:** 10.3390/jpm13121669

**Published:** 2023-11-29

**Authors:** Vyacheslav V. Ryabov, Darya A. Vorobeva, Irina V. Kologrivova, Tatiana E. Suslova

**Affiliations:** Cardiology Research Institute, Tomsk National Research Medical Center, Russian Academy of Sciences, 634012 Tomsk, Russia; rvvt@cardio-tomsk.ru (V.V.R.); ikologrivova@gmail.com (I.V.K.); tes@cardio-tomsk.ru (T.E.S.)

**Keywords:** non-obstructive coronary artery atherosclerosis, myocardial infarction with non-obstructive coronary arteries, inflammation, endothelial dysfunction, cytokines

## Abstract

The objective of our study was to evaluate the concentrations of pro-inflammatory biomarkers in patients with acute myocardial infarction with non-obstructive coronary arteries (MINOCA) compared to patients with acute myocardial infarction with obstructive coronary arteries (MI-CAD) in the early post-infarction period and after 1 year and to perform a comparative analysis of the relationship between laboratory biomarkers and atherosclerosis progression in patients with MINOCA and MI-CAD. Methods: Samples of peripheral venous blood were collected upon admission and on days 2, 4, and 7 of hospitalization and after 1 year. An extended multiplex analysis was performed in blood serum. Multidetector-computed tomography coronary angiography was performed on day 7 and 1 year after acute myocardial infarction to assess the progression of atherosclerosis. Results: The level of high-sensitive C-reactive protein (hsCRP) was elevated upon admission in MINOCA patients compared to MI-CAD patients (*p* = 0.05), but it was comparable in two groups at other time points and did not exceed the reference range after 1 year. Despite comparable levels of cytokines CXCL-6, LIGHT, CCL-8, and endocan-1 in patients in both groups, MINOCA patients had a greater increase in pro-inflammatory cytokines PlGF, oncostatin M, IL-20, and CCL-15 sVCAM-1 in the early post-infarction period and in CCL-21, sVCAM-1, oncostatin M, and PlGF after 1 year. We observed significant differences in the dynamics of the following biomarkers between patients with MI-CAD and MINOCA: the dynamics of concentrations of CCL21 (*p* = 0.002), LIGHT (*p* = 0.03), and endocan-1 (*p* = 0.03) after 1 year compared to day 1 in MI-CAD and MINOCA patients was opposite, while the dynamics of CXCL6 (*p* = 0.04) and endocan-1 (*p* = 0.02) differed between groups when evaluated after 1 year compared to day 7 of the early post-infarction period. In the MINOCA group, factors associated with atherosclerosis progression were concentrations of sVCAM-1 and CCL-21, while in the MI-CAD group, concentrations of CCL-8 and CXCL6 were the main determinants of atherosclerosis progression. Conclusions: This small study showed that MINOCA and MI-CAD patients exhibited differences in a pro-inflammatory biomarker profile in the early post-infarction period and after 1-year follow-up, which implies distinct inflammatory pathways involved in atherogenesis during MINOCA. The key factors that were associated with atherosclerosis progression in MINOCA patients are sVCAM-1 and CCL-21, which may suggest a complex genesis of atherosclerosis progression due to structurally altered plaques and changes in the microcirculatory bed. In MI-CAD patients, CCL-8 and CXCL-6 were the key biomarkers associated with atherosclerosis progression. Further large-scale studies are required to confirm our data.

## 1. Introduction

Over the past decade, progress in myocardial revascularization and aggressive lipid -lowering and antithrombotic therapy has increased the frequency of the recurrent ischemic events after acute myocardial infarction (AMI) [1]. However, despite the achieved target values of lipids and high sensitive C-reactive protein (hsCRP) in some patients after AMI, the progression of coronary atherosclerosis is very frequently observed both in patients with AMI with obstructive coronary arteries (MI-CAD) and in patients with non-obstructive coronary arteries (MINOCA) [2,3].

Therefore, it is necessary to search for new laboratory markers associated with the progression of coronary atherosclerosis, keeping in mind that the underlying mechanisms of atherogenesis might be different depending on the presence or absence of the coronary obstruction.

Numerous studies have been devoted to the evaluation of pro- and anti-inflammatory factors based on multiplex panels [4,5]. These panels are allowed not only to measure a large number of laboratory biomarkers but identify various pathogenetic mechanisms underlying the course of myocardial infarction at different periods, as well as the progression of atherosclerosis.

Notwithstanding the increasing interest in MINOCA patients, studies of this group of patients are mainly focused on instrumental approaches of diagnostics, for instance, optical coherence tomography, intravenous ultrasound, and cardiac magnetic resonance imaging [6]. Studies on laboratory biomarkers in MINOCA patients are very scarce. For instance, several studies have evaluated biomarkers during the acute phase of MINOCA, mainly focusing on the biomarkers of myocardial necrosis and hsCRP [6,7]. Hjort M. et al. (2019) studied pro-inflammatory cytokines and showed that MINOCA patients could exhibit a greater pro-inflammatory predisposition compared to MI-CAD patients [8]. The analysis of cytokines in this study was carried out only 3 months after the ischemic event, when patients had already received the prescribed medication quite a long time previously, which could have mitigated the severity of aseptic inflammation.

Data on the dynamics of pro-inflammatory biomarkers in the early post-infarction period and after 1 year in MINOCA and MI-CAD patients are currently not available. In view of the heterogeneity of MINOCA patients and matching endpoints compared to patients with obstructive coronary arteries, it is important to conduct a multi-marker laboratory comparison in order to evaluate pathological features in these two groups, which may presumably be different, as well as to assess the relationship with the progression of coronary atherosclerosis.

The objective of this study was to evaluate the concentrations of pro-inflammatory biomarkers in patients with MINOCA and patients with MI-CAD in the early post-infarction period and after 1 year, and to perform a comparative analysis of the relationship between laboratory biomarkers and atherosclerosis progression in the patients of both groups.

## 2. Materials and Methods

Non-randomized, open, and controlled study was performed. The study is registered on ClinicalTrials.gov: NCT03572023 (date of registration 28 June 2018) and contains the study protocol. The study was conducted according to the principles of the Declaration of Helsinki and was approved by the hospital Human Research Ethics Committee of the Research Institute of Cardiology, Tomsk National Research Medical Center, protocol No. 164 of 23 November 2017. All patients were consistently admitted and included in the study in 2017–2018 after the provision of the voluntary informed consent.

The inclusion criteria for the MINOCA group were as follows: patients (18 years old and older) with acute coronary syndrome (ACS), who underwent coronary angiography within 24 h after disease onset, with non-obstructive (≤50%) coronary atherosclerosis evidenced by the invasive coronary angiography results, high and moderate cardiovascular risk on the GRACE scale, and sinus rhythm.

The exclusion criteria for the MINOCA group were contraindications to adenosine administration, hemodynamic instability, myocardial inflammatory diseases, moderate-to-severe cardiac valvular disease, atrial fibrillation, previous revascularization, severe comorbidity, severe renal failure (estimated glomerular filtration rate < 30), pacing, and claustrophobia. Patients with Takotsubo syndrome were not included in this study.

**Figure 1:**
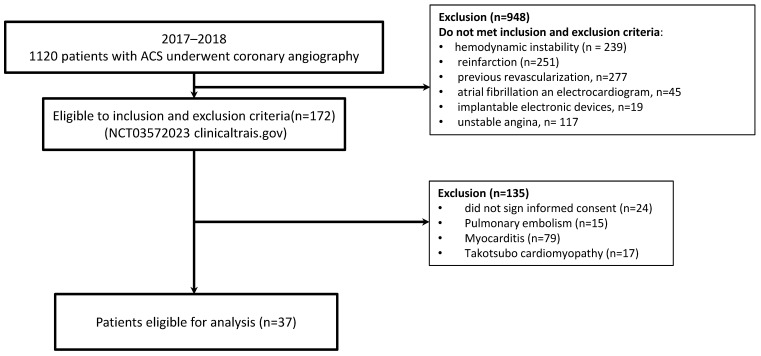
Flow chart showing patients included in the study. ACS, acute coronary syndrome.

The inclusion criteria for the MI-CAD group were as follows: patients (18 years old and older) with ACS, who underwent invasive coronary angiography within 24 h after disease onset, with stenosis ≥ 75% of one coronary artery, high and moderate cardiovascular risk on the GRACE scale, and sinus rhythm. The exclusion criterion for this group was myocardial infarction associated with revascularization; other criteria were similar to those of the MINOCA group. Figure 1 presents the flow chart showing patients included in the study. Standard echocardiography was performed on day 4 using a VIVID E9 ultrasound system (GE Healthcare).

### 2.1. Invasive Coronary Angiography

All patients underwent quantitative coronary arteriography performed using an Axiom Artis coronary angiography system (Siemens; Erlangen, Germany). Invasive coronary angiography (ICA) in patients was performed using a 5F Judkins-type catheter through the femoral access. The coronary artery stenoses were quantitatively assessed using dedicated software by two experienced researchers. Coronary artery stenosis of ≥50% in major epicardial coronary arteries and in the left main coronary artery was considered significant. The criterion to define the slow coronary flow was a corrected frame count greater than 2 standard deviations from the normal range (21 ± 3) [9].

### 2.2. Multidetector-Computed Tomography Coronary Angiography Protocol 

Multidetector-computed tomography coronary angiography (MDCT-CA) was performed on day 7 and 1 year after acute myocardial infarction to assess the progression of atherosclerosis.

During MDCT-CA, all patients had a sinus rhythm with a heart rate of 50–65 beats per minute. The heart rate and blood pressure were evaluated before each scan. Patients with a heart rate higher than 60 bpm were treated with an intravenous infusion of 1 mg metoprolol before CT scan, and all patients received 0.5 mg of sublingual nitroglycerin. MDCT-CA was performed using a 64-detector CT scanner (GE Discovery NM/CT 570c, GE Healthcare, Milwaukee, WI, USA).

An unenhanced scan of coronary artery calcium scoring was obtained according to the following protocol: prospective triggering at 75% of R-R interval; tube voltage of 120 kV; tube current of 400 mA; and 1.25 mm slice thickness.

For the contrast-enhanced scans, 70–90 mL of nonionic contrast agent (Iopamidol 370 mg iodine/mL, Bracco Diagnostics, Milano, Italy) was injected intravenously through an 18-gauge antecubital catheter at a flow rate of 5–5.5 mL/s followed by 40 mL saline injection. The obtained data were reconstructed in the diastole phase (mostly, 75% of RR interval duration) and analyzed using Advantage Workstation 4.6, GE Healthcare [10].

An increase in stenosis of 10% or more was recognized as the progression of atherosclerosis. The total radiation exposure ranged from 4 to 5.5 mSv.

### 2.3. Biochemical Analysis

Samples of peripheral venous blood were collected into clotting activator tubes (BD, Franklin Lakes, NJ, USA) upon admission; on days 2, 4, and 7 of hospitalization; and after 1-year follow-up.

Troponin I was detected using the AccuTanE test system manufactured by Beckman Coulter (Diagnostics, Brea, CA, USA). The 99th percentile from the upper reference level (cTnI laboratory reference cut-off for normalcy: <0.04 ng/mL) was conventionally taken as a threshold to diagnose myocardial injury.

The lipid profile was analyzed in blood serum upon admission using the enzyme-colorimetric method (Diakon, Pushchino, Russia) and included the detection of serum concentration of total cholesterol, triacylglycerol, and high-density lipoprotein (HDL) cholesterol (Diakon, Pushchino, Russia). The concentration of low-density lipoprotein (LDL) cholesterol was calculated using the formula [LDL] = [Total cholesterol] − [Triglycerides (TG)]/2.2 − [HDL].

The remaining blood serum samples were aliquoted in plastic tubes and stored at −40 °C until the final analysis.

The concentration of hsCRP was detected using the enzyme-linked immunosorbent assay (VECTOR-BEST, Novosibirsk, Russia) using the Infinite F500 microplate reader (Tecan, Männedorf, Switzerland) and Magellan software (Tecan, Männedorf, Switzerland).

An extended multiplex analysis was performed. In blood serum, the concentration of the following indicators was determined: endocan-1, oncostatin M, placental growth factor (PlGF), chemokine ligands 6 (CXCL6), tumor necrosis factor ligand (LIGHT), soluble P-selectin (sP-Selectin), sVCAM-1 (soluble vascular cell adhesion molecule-1), interleukin 20 (IL-20), CCL-8 (MCP-2, monocyte chemotactic protein-2), CCL-15 (leukotactin-1), and CCL-21 (6Ckine/Exodus-2). All the parameters were analyzed using a Multiplex Instrument FLEXMAP 3D system (Luminex Corporation, Austin, TX, USA); MILLIPLEX map Human Cardiovascular Disease Panel 1 and 2; cytokines/chemokines Panel 2; and MILLIPLEX Analyst 5.1 software (Merck KGaA, Milliplex, Darmstadt, Germany). A routine complete blood count was performed using the automatic hematological analyzer.

### 2.4. Statistical Analysis

A statistical analysis was performed using StatTech v. 3.1.3 (StatTech LLC, St. Petersburg, Russia). The distribution of continuous variables was evaluated via the Shapiro–Wilk W-test. Continuous variables were expressed as median with quartiles (Q25–Q75). For categorical variables, the results were reported as absolute counts (n) and percentages (%). Nominal data were analyzed using Pearson’s χ^2^ test and two-sided Fisher’s exact test (at expected frequencies less than 5). Since the distribution of the studied parameters was different from normal according to the results of the Shapiro–Wilk test, the nonparametric Mann–Whitney U-test was used to compare continuous variables in two independent groups. A Wilcoxon test was used to compare changes in the parameters at different time points in the same group (in the early post-infarction period and after 1 year). To evaluate the dynamics of indicators, the following calculations were performed: Delta 1—the difference between the indicators after 1 year and 1 day; Delta 2—the difference between the indicators after 1 year and 7 days.

A prognostic model for the probability of a binary outcome was developed using logistic regression. Nagelkerke R^2^ was used as a measure of the model performance.

The accuracy of atherosclerosis progression detection was assessed via receiver operator characteristic (ROC) analysis, reporting areas under the curve (AUC) and their associated 95% confidence intervals. The best values in the prediction of atherosclerosis progression were defined as the cut-off point with the highest Youden index. The *p* ˂ 0.05 value was considered statistically significant.

## 3. Results

### Baseline Characteristics

A total of 37 patients with myocardial infarction were enrolled in the study: 16 patients constituted the main group (MINOCA) and 21 patients were included in the MI-CAD group.

The differences in demographic, anamnestic, and clinical characteristics were determined by gender, history of angina, time of hospital admission, the GRACE risk score, thrombolytic therapy at the prehospital stage, TIMI 2 flow detected via invasive coronary angiography, and the presence of acute left ventricle apical aneurysm with parietal thrombosis. The influence of gender on the development of myocardial infarction in MINOCA patients was not revealed. The main clinical and anamnestic data for MINOCA and MI-CAD patients are summarized in Table 1.

Upon admission and after 1 year, all patients received standard therapy for acute coronary syndrome according to the national recommendations; patients in both groups received dual antiplatelet and lipid-lowering therapy (100%); 14 (87.5%) patients in the main group and 20 (95.2%) patients in the control group received beta-blockers; 12 (75.0%) patients in the main group and 20 (95.2%) patients in the control group received angiotensin-converting enzyme inhibitors; calcium channel blockers were prescribed to 7 (36.8%) patients in the main group and 1 (4.7%) patient in the control group; and unfractionated heparin was given to 11 (68.7%) patients in the main group and 19 (90.4) patients in the control group, with a subsequent switch to low molecular weight heparins.

**Figure 2:**
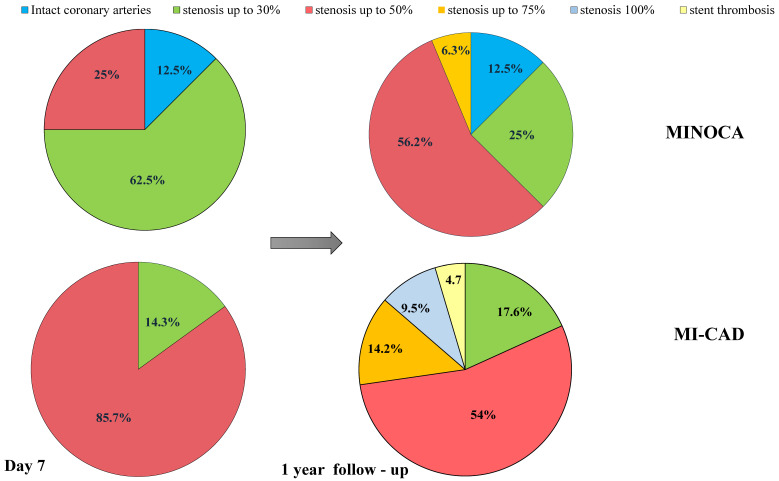
The ratio of plaque structure on day 7 after myocardial infarction and after 1 year in both groups.

After 1-year follow-up, according to MDCT-CA in MINOCA patients, the proportion of patients with 30% stenosis decreased, and the proportion of patients with 50% stenosis increased (*p* = 0.004). Of note, one patient from the MINOCA group experienced the progression of atherosclerosis of up to 70%. Similar data were obtained in the MI-CAD group, where stenosis (up to 70%) was detected in 14.3% of patients, and occlusion (100%) was detected in 9.5% of patients. In the MI-CAD group, restenosis and stent thrombus were detected in two patients (Figure 2). The progression of atherosclerosis was observed in MINOCA patients with higher frequency compared to MI-CAD patients, even though these differences did not attain statistical significance levels: 10 (62.5%) patients with MINOCA vs. 10 (47.6%) patients with MI-CAD (*p* = 0.21).

MINOCA patients, as expected, showed lower levels of troponin I at on days 2, 4 and 7 upon admission. Meanwhile, the concentration of hsCRP in the MINOCA group significantly exceeded that in the MI-CAD group upon admission and remained higher throughout the period of observation even though it did not reach statistical significance levels (Table 2). After 1 year, despite lower concentrations of hsCRP and LDL/HDL in both groups, the total cholesterol level in the MINOCA group did not change and was higher than that in the MI-CAD group (Table 2).

Indicators of the multiplex analysis of blood serum in patients of the studied groups are summarized in Table 3. The levels of CXCL6 and LIGHT did not significantly change in both studied groups throughout the entire follow-up period and remained comparable between patients both with and without coronary obstruction (Table 3).

Unlike MI-CAD patients, MINOCA patients had higher concentrations of CCL-21 after 1-year follow-up, placental growth factor (PlGF) on days 1, 2, and 7 and after 1 year; oncostatin M on days 4 and 7 and after 1 year; IL-20 on days 2 and 4; CCL-15 on days 1, 2, 4, and 7; and sVCAM-1 on day 1 and after 1 year (Table 3). Concentrations of sP-selectin on days 1 and 2 were lower in MINOCA patients compared to MI-CAD patients, but these parameters became comparable after 1-year follow-up (Table 3).

In MINOCA patients, parameters that significantly changed after 1-year follow-up were as follows: concentrations of CCL-21 (*p* = 0.04) increased, while the concentration of endocan-1 (*p* = 0.003) decreased. In MI-CAD patients, there was a significant decrease in the concentration of endocan-1 (*p* = 0.004). Other parameters did not change significantly within the groups (*p* > 0.05) (Table 3). Time curves of the concentrations and dynamics of laboratory biomarkers of the studied groups are represented in Supplementary Figures S1 and S2.

The dynamics of laboratory parameters depending on the presence of coronary obstruction was also assessed. Significant differences in the dynamics of the following biomarkers were observed between patients with MI-CAD and MINOCA: the dynamics of the concentrations of CCL21, LIGHT, endocan-1, and sVCAM-1 (*p* = 0.03) were different after 1 year compared to day 1 during MI-CAD and MINOCA, while the dynamics of CXCL6 and endocan-1 were different after 1 year compared to day 7 of the early post-infarction period (Table 4).

A logistic regression analysis was used to evaluate potential biomarkers contributing to the progression of atherosclerosis in each of the studied groups. For the analysis, we included parameters that increased after 1 year, according to Table 3 and Table 4, or significantly differed between the groups. Characteristics of the association of predictors with the probability of atherosclerosis progression are presented in Table 5 and Table 6.

According to the predictive model of binary logistic regression, factors associated with the progression of atherosclerosis in MINOCA patients included concentrations of sVCAM-1 (day 7) and CCL-21 (day 7). The observed association was described using the following equation:

Progression of atherosclerosis = 1/(1 + e^−z^) × 100%

z = −1.928 + 0.008X_CCL-21_ − 0.006X_sVCAM-1_

**Figure 3:**
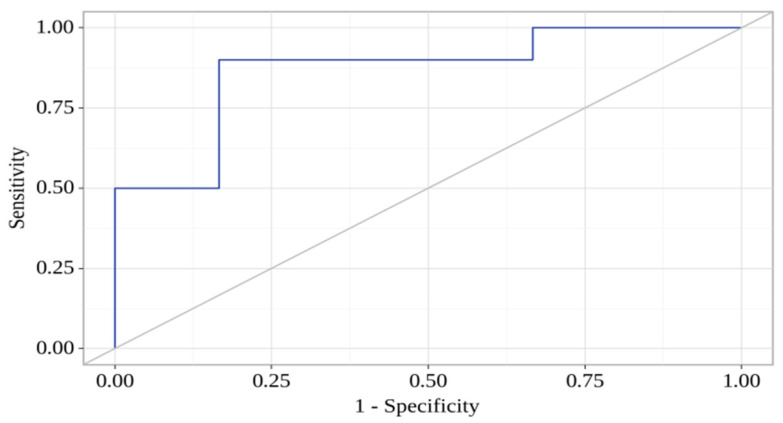
ROC curve characterizes the dependence of the probability of atherosclerosis progression on CCL-21 and sVCAM-1 concentrations.

The resulting regression model was statistically significant (*p* < 0.001). Based on the signs of the regression coefficients, a direct relationship of sVCAM-1 and CCL-21 with the probability of atherosclerosis progression was established. The area under the ROC curve comprised 0.867 ± 0.063 with 95% CI: 0.742–0.991 (Figure 3). The sensitivity and specificity of the model were 90.0% and 83.3%, respectively.

We used a step-by-step inclusion of laboratory biomarkers in the analysis of binary logistic regression for the MI-CAD group to identify factors associated with the progression of atherosclerosis. We found that atherosclerosis progression in MI-CAD patients was related to concentrations of CCL-8 (day 7) and CXCL6 (day 7). The observed association could be described using the following equation:

Progression of atherosclerosis = 1/(1 + e^−z^) × 100%

z = 3.349 + 0.070X_CCL-8_ − 0.024X_CXCL-6_

**Figure 4:**
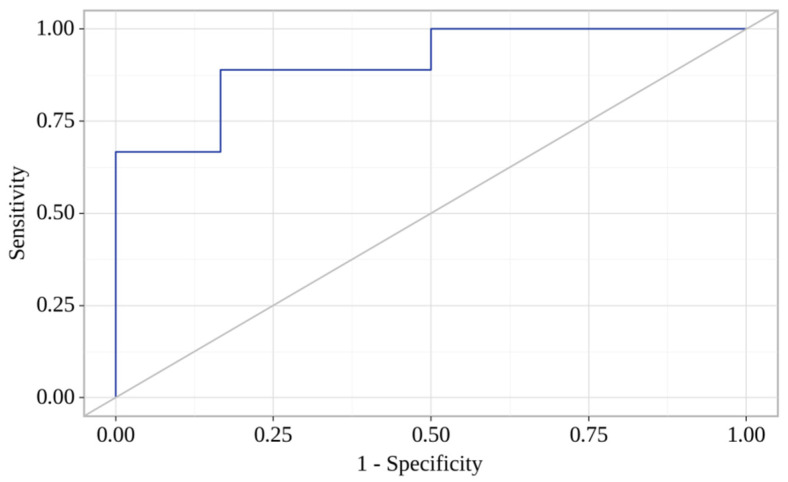
ROC curve characterizes the dependence of the probability of atherosclerosis progression on CCL-8 and CXCL-6 concentrations.

The resulting regression model was statistically significant (*p* = 0.001). Based on the signs of the regression coefficients, a direct relationship between the levels of CCL-8 (day 7) and CXCL-6 (day 7) with the progression of atherosclerosis probability was established. Characteristics of the association of predictors with the probability of atherosclerosis progression are presented in Table 6. The area under the ROC curve comprised 0.907 ± 0.055 with 95% CI: 0.800–1.000 (Figure 4). The sensitivity and specificity of the method were 88.9% and 83.3%, respectively.

## 4. Discussion

For the first time, we extensively studied and compared concentrations of pro-inflammatory biomarkers in MINOCA and MI-CAD patients in the early post-infarction period and after 1 year. We demonstrated for the first time that the relationships between concentrations of laboratory biomarkers and the progression of atherosclerosis in MINOCA patients differ from those in MI-CAD patients.

This study reports that MINOCA patients had lower levels of troponin I and the wall motion score index. This suggests less myocardial damage in MINOCA patients compared to that in MI-CAD patients, as demonstrated in our previous work [11,12].

We compared the concentrations of pro-inflammatory cytokines and found comparable changes in the content of cytokines CXCL-6, LIGHT, and CCL-8, which may be indicative of similar mechanisms occurring during atherosclerosis in both MI-CAD and MINOCA patients. Meanwhile, statistically significant differences between MI-CAD and MINOCA groups were determined for the following biomarkers: PlGF, oncostatin M, sP-Selectin, LIGHT, IL-20, CCL-15, CCL-21, sVCAM-1, and endocan-1.

CCL-15 is a pro-inflammatory cytokine, a chemotactic factor involved in the recruitment of leukocytes into the arterial wall. According to our results, the CCL-15 concentration was significantly higher in MINOCA patients in the early post-infarction period and after 1 year compared to MI-CAD patients. CCL-15 was shown to contribute to plaque destabilization in the progression of subclinical atherosclerosis [13]. According to V. Sucato et al., optical coherence tomography was employed to show that MINOCA patients exhibit infarct-related plaques with a lipid-rich body and thin fibrous cap, which makes them vulnerable to rupture [14]. In addition, CCL-15 increases the prothrombotic activity by inducing tissue factor expression [11], which may explain an increase in prothrombotic activity in MINOCA patients [15]. After 1 year, the CCL-15 concentration decreased, probably due to the stabilization of plaques and the lipid-lowering therapy.

Similar to CCL-15, sP-Selectin is a pro-inflammatory cytokine responsible for atherothrombosis. It represents an integral membrane glycoprotein of platelets and endothelial cells [16]. The plasma sP-selectin level is elevated in AMI, and it increases further after thrombolytic therapy. This increase is probably induced by activation of endothelial cells or platelets after myocardial ischemia and reperfusion during AMI [17]. In our study, atherothrombosis obviously took place in both groups. However, AMI with ST elevation was more common among MI-CAD patients, and thrombolytic therapy was performed more often in that group [17]. This may explain a greater increase in this indicator (on days 1 and 2) in MI-CAD patients. The increased level of sP-Selectin after 1 year in MINOCA patients indicates the residual elevated platelet activation, as was shown previously [11,14].

Another pro-inflammatory cytokine with an atherogenic effect that causes microvascular lesions due to the induction of angiogenesis in hypoxic tissue is IL-20. In AMI, it promotes cardiomyocyte apoptosis and increases myocardial reperfusion injury [18,19]. In addition, there is evidence that cardiomyocyte necrosis in ischemic myocardium contributes to the release of pro-inflammatory cytokines and hsCRP [20]. The concentrations of Troponin I were higher and, consequently, the infarct size was larger in MI-CAD patients, which evokes a contradiction between the identified hyperproduction of pro-inflammatory cytokines (IL-20) and a lower level of necrosis in MINOCA patients. In our opinion, this phenomenon can be attributed to a higher production of pro-inflammatory cytokines in MINOCA patients due to more active inflammatory processes in the atherosclerotic plaque or the vessel wall. Our data are consistent with those reported by M. Hjort [8]. However, in the study by M. Hjort et al., hsCRP in MINOCA patients was lower than that in MI-CAD patients. In our study, the level of hsCRP was higher in MINOCA patients on day 1 in the early post-infarction period (Table 2), probably due to more severe inflammation.

In addition, we found an increased concentration of pro-inflammatory active chemokine CCL-21 in MINOCA patients after 1 year. Unlike IL-20, CCL-21 does not reflect the degree of myocardial damage, but it indicates plaque progression and instability, as well as CCL-15, which is significantly elevated in MINOCA compared to MI-CAD patients in the early post-infarction period [21,22]. Damas J.K. et al. showed that CCL-21 might contribute to atherogenesis via different mechanisms including the enhanced recruitment of T cells and macrophages to atherosclerotic lesions [23]. The increased concentrations of CCL-21 and LIGHT after 1-year follow-up in MINOCA patients compared to the decreased concentrations of the above indicators in MI-CAD patients (Table 3) may indicate ongoing inflammation in the vessel walls during MINOCA [24,25]. This hypothesis is supported by the fact that the target values of the lipid spectrum were not achieved in the MINOCA group in contrast to the MI-CAD group (Table 2), which may be due to less aggressive lipid-lowering therapy. Statins have pleotropic effects and may reduce inflammation in vessel wall in MI-CAD patients [26,27], but the effect of statins on inflammation in MINOCA patients requires further studies to be conducted in the future.

Other indicators that were considered in this study and appeared to be different in MI-CAD and MINOCA patients are endocan-1 and sVCAM-1 (Table 3 and Table 4). Serum levels of endocan-1 and sVCAM-1 indicate endothelial dysfunction in AMI patients. An ischemic event causes not only myocardial necrosis but also endothelial cell damage, which results in the release of endocan-1 and sVCAM-1 [28,29]. Taking into account a higher concentration of sVCAM-1 and a more pronounced dynamics of endocan-1 in MINOCA patients, we assume that MINOCA patients had more pronounced endothelial dysfunction. A significantly higher concentration of endocan-1 after 1 year can be attributed to a partial restoration of endothelial function after AMI, and the preservation of sVCAM-1 elevated values can be due to a chronic aseptic inflammation in MINOCA patients with a slow coronary flow in comparison with MI-CAD patients.

Compared to MI-CAD patients, the observed changes are in accordance with a greater increase in PlGF level in the early post-infarction period and after 1 year in MINOCA patients. PlGF, a pro-inflammatory cytokine and a member of the vascular endothelial growth factor family of angiogenic proteins, plays an important role in pathological angiogenesis. It enhances endothelial activation and macrophage recruitment, induces tissue repair, and improves cardiac performance via the recruitment of bone marrow-derived progenitor cells to infarcted myocardial tissue [30,31]. Its release during ACS takes place in response to the endothelial damage and aseptic inflammation, which most likely leads to a statistically greater increase in PlGF in MINOCA patients.

Oncostatin M is a pleiotropic cytokine of the interleukin IL-6 family, with complex functions in atherogenesis. Oncostatin M can stimulate the production of P-selectin and sVCAM-1 by endothelial cells and induce the angiogenesis of microvascular endothelial cells [32]. Patel P. et al. showed that oncostatin M is one of the initiating molecules in atherosclerosis progression [33]. Hence, a significant increase in this indicator may explain increased levels of sVCAM-1 in MINOCA patients and is likely to represent an upstream regulator of plaque formation, progression, and vulnerability.

The logistic analysis revealed that sVCAM-1 (on day 7) and CCL-21 (on day 7) were associated with the progression of atherosclerosis in MINOCA patients. In the MI-CAD group, concentrations of CCL-8 (on day 7) and CXCL-6 (on day 7) were associated with the progression of atherosclerosis. This presumably indicates different mechanisms that lead to the progression of atherosclerosis in each group.

Based on these logistic analysis data, we hypothesize that the progression of coronary atherosclerosis in MINOCA patients is not only due to the influence of pro-inflammatory cytokines but also due to microcirculatory impairment manifested by endothelial dysfunction. sVCAM-1 is one of the indicators of endothelial dysfunction, which is released during the loss of protective properties of the endothelium. Endothelial dysfunction is associated with the damage of endothelial junctions and an increase in the permeability of endothelium to macromolecules [34]. These changes cause a subendothelial accumulation of cholesterol-containing lipoproteins, which triggers a low-grade inflammatory response and atherosclerosis progression [34,35]. Recio-Mayoral A. et al. showed a correlation between the increased concentration of CRP, decreased coronary blood flow reserve, chronic inflammation, and atherosclerosis in patients with stable angina and non-obstructive coronary arteries [36], which was discussed in a position paper on coronary microvascular dysfunction in MINOCA patients [37].

In addition, microvascular dysfunction has been shown to be associated with the development of heart failure with preserved ejection fraction, which is associated with increased expression of proinflammatory cytokines [38,39]. The identification of such proinflammatory cytokines may be important, as targeting inflammatory mediators is a promising direction in the therapy of heart failure and atherosclerosis progression.

Unfortunately, we did not specifically evaluate the presence of microvascular dysfunction, similarly to another research group, which evaluated the concentration of pro-inflammatory cytokines three months after the index event [8]. However, with regard to the fact that 56% of MINOCA patients had a slow coronary blood flow according to ICA (TIMI 2) (Table 1), we cannot reject the hypothesis of a complex influence of structurally altered plaques and changes in the microcirculatory bed on the progression of atherosclerosis in MINOCA patients.

In contrast to MINOCA patients, the logistic regression model of the probability of atherosclerosis progression in MI-CAD patients included CCL-8 and CXCL-6, which are pro-inflammatory cytokines that play an important role in atherogenesis [40,41]. Despite the target lipid profile values being achieved, patients showed the progression of atherosclerosis. Therefore, these cytokines can serve as markers of the residual inflammatory risk in this group and can help to identify patients who require more careful monitoring and aggressive treatment. Further large-scale studies are needed to confirm our data.

Based on these data, we consider molecular phenotyping to be a very promising tool for laboratory diagnostics in MINOCA patients to reveal fundamental mechanisms that affect atherosclerosis progression. In the future, it may have major clinical implications for managing these patients and improving prognosis.

## 5. Conclusions

This small study revealed that MINOCA and MI-CAD patients showed differences in pro-inflammatory biomarkers in the early post-infarction period and after 1-year follow-up. Despite the comparable levels of cytokines CXCL-6, LIGHT, CCL-8, and endocan-1 in patients of both groups, MINOCA patients exhibited a greater increase in pro-inflammatory cytokines PlGF, oncostatin M, IL-20, and CCL-15 sVCAM-1 in the early post-infarction period and CCL-21, sVCAM-1, oncostatin M, and PlGF after 1 year. MI-CAD patients showed a greater increase in sP-Selectin in the early post-infarction period. The key biomarkers that were associated with atherosclerosis progression in MINOCA patients are sVCAM-1 and CCL-21, which may suggest a complex genesis of atherosclerosis progression due to the structurally altered plaques and changes in the microcirculatory bed. In MI-CAD patients, CCL-8 and CXCL-6 were the key biomarkers associated with atherosclerosis progression. Further large-scale studies are needed to confirm our data.

## 6. Limitations

This study was conducted as a single-center trial. The main limitation of our study is a small number of patients being recruited due to the strict inclusion criteria. Out of 1120 patients who underwent coronary angiography, only 37 were eligible for the final study. Additionally, since the presence of atrial fibrillation was an exclusion factor from the study, due to the potential impact to the analyzed parameters, our findings cannot be extrapolated to AMI patients with atrial fibrillation. The obtained results may also not be extrapolated to populations of different ethnic backgrounds. The influence of gender on the development of myocardial infarction was not evaluated either. Meanwhile, we managed to perform a thorough examination of patients and revealed the most promising biomarkers that should be evaluated in future large-scale trials. The case-control design of our work did not allow us to define the prognostic value of the evaluated biomarkers. The approximation of the markers in the whole cohort cannot be evaluated or approximated using statistical methods to assess the potential prognostic significance for the progression of coronary atherosclerosis on a small sample; therefore, it should be carried out on a larger sample of patients.

## Figures and Tables

**Table 1:** Clinical, anamnestic characteristics and laboratory blood test results of patients.

Number of Patients, n%	MINOCA, n = 16	MICAD, n = 21	*p*-Value
Men, n (%)	7 (43.7)	17 (80.9)	0.02
Age, mean (Q25; Q75)	66.0 (54.71)	60 (56; 68)	0.33
Hypertension, n (%)	13 (81.3)	16 (76.1)	0.71
Dyslipidemia, n (%)	14 (87.5)	17 (80.9)	0.89
Overweight, n (%)	4 (25)	11 (52.3)	0.15
Family history of CAD, n (%) *	7 (43.7)	13 (61.9)	0.27
Smoking, n (%)	5 (31.3)	11 (52.3)	0.26
Diabetes mellitus, n (%)	0	4 (19.0)	0.02
GFR, ml/min/1.73 m2, mean (Q25; Q75)	71.5 (54.0; 80.0)	79.0 (65.0; 89.0)	0.20
History of angina pectoris, n (%)	10 (62.5)	6 (28.5)	0.04
History of stroke, n (%)	1 (5.2)	2 (9.5)	0.71
Peripheral atherosclerosis, n (%)	4 (25)	7 (33.3)	0.58
Time of admission to the hospital, min, mean (Q25; Q75)	390 (146.5; 870)	180 (98; 240)	0.02
STEMI, n (%)	10 (62.5)	19 (90.4)	0.01
GRACE, risk, mean (Q25; Q75)	2.0 (2.0; 3.5)	2.3 (2.0; 5.0)	0.26
Thrombolytic therapy, n (%)	3 (18.7)	11 (52.3)	0.007
TIMI 2 flow, n (%)	9 (56.3)	1(4.7)	0.01
Wall motion score index, score	1.0 (1.0; 1.2)	1.2 (1.2; 1.5)	0.04
Left ventricular ejection fraction, %	60.0 (45.0; 60.0)	56.0 (50.0; 60.0)	0.51
Acute apical left ventricle aneurysm, n (%)	3 (18.7)	2 (9.5)	0.62

Note: * burdened heredity for cardiovascular pathology; GFR—glomerular filtration rate; STEMI—ST-segment elevation myocardial infarction.

**Table 2:** Laboratory parameters of blood.

Indicator (Reference Range)	Day	MINOCA, n = 16	MI-CAD, n = 21	*p*-Value
Troponin I ng/mL (0.00–0.040)	2	0.5 (0.11; 8.3)	4.9 (1.0; 25.2)	0.02
	4	0.4 (0.07; 1.7)	0.7 (0.5; 4.4)	0.04
	7	0.08 (0.02; 0.2)	0.4 (0.2; 0.9)	0.0003
	1 year	0.01 (0.01; 0.02)	0.01 (0.01; 0.02)	0.50
Cholesterol, mmol/L (<4.5)	1	4.8 (4.2; 6.2)	4.5 (3.9; 4.9)	0.17
	1 year	4.4 (3.6; 5.7)	3.6 (2.9; 4.3)	0.01
Triglycerides, mmol/L (0.5–1.7)	1	1.4 (0.9; 2.5)	1.7(1.1; 2.0)	0.88
	1 year	1.1 (0.7; 1.7)	1.2 (0.7; 1.6)	0.84
HDL-C, mmol/L (>1.0)	1	1.2 (0.9; 1.5)	1.1 (1.1; 1.3)	0.58
	1 year	1.4 (1.2; 1.8)	1.1 (0.9; 1.2)	0.01
LDL-C, mmol/L (<2.5)	1	2.7 (2.3; 4.0)	2.6 (2.2; 2.8)	0.23
	1 year	2.4 (1.5; 3.8)	1.5 (1.3; 2.1)	0.12
LDL/HDL (<2.5)	1	2.8 (1.6; 3.0)	2.1 (1.8; 2.3)	0.39
	1 year	1.7 (0.78; 2.6)	1.48 (0.9; 2.3)	0.04
hsCRP, mg/L (<3.0)	1	16.5 (3.8; 30.0)	4.4 (3.8; 5.0)	0.04
	4	14.0 (4.8; 18.7)	4.7 (3.9; 12.8)	0.11
	7	5.3 (3.3; 10.0)	4.0 (3.5; 13.1)	0.84
	1 year	3.7 (2.8; 10.1)	3.1 (2.0; 4.0)	0.23

Note: hsCRP—high-sensitivity C-reactive protein; HDL-C—high-density lipoprotein cholesterol; LDL-C—low-density lipoprotein cholesterol.

**Table 3:** Indicators of the multiplex analysis of blood serum in patients of the studied groups.

Indicator (Reference Range)	Day	MINOCA, n = 16	MI-CAD, n = 21	*p*-Value
CXCL6, pg/mL, Me (Q25; Q75)	1	247.33 (213.34; 281.89)	224.05 (167.99; 315.45)	0.57
	2	218.81 (201.17; 229.00)	244.35 (201.31; 289.62)	0.41
	4	236.81 (219.44; 252.11)	218.88 (175.84; 246.74)	0.26
	7	242.97 (226.40; 288.77)	229.03 (214.07; 275.94)	0.41
	1 year	227.28 (208.75; 271.70)	270.00 (147.70; 301.64)	0.53
CCL-8, pg/mL, Me (Q25; Q75)	1	45.9 (41.2; 56.9)	40.3 (28.3; 47.1)	0.49
	2	42.7 (43.1; 53.8)	39.5 (36.1; 45.2)	0.06
	4	44.9 (40.8; 56.4)	44.8 (39.6; 47.5)	0.49
	7	39.8 (27.1; 63.3)	38.9 (451; 54.4)	0.44
	1 year	47.1 (40.8; 64.4)	49.5 (40.3; 52.3)	0.54
CCL-15, pg/mL, Me (Q25; Q75)	1	4931.0 (3317.5; 7496.5)	3022.5 (2006.0; 4574.0)	0.04
	2	5683.5 (3284.0; 7168.0)	3500.0 (2901.0; 4098.0)	0.02
	4	5261.0 (3355.0; 6171.0)	2606.0 (2389.0; 3851.0)	0.04
	7	4906.5 (4199.5; 5733.5)	3732.0 (2653.0; 3951.0)	0.02
	1 year	3694.0 (2623.5; 5428.0)	2657.0 (2154.0; 3319.0)	0.19
CCL-21, pg/mL, Me (Q25; Q75)	1	96.9 (38.4; 192.9)	193.8 (165.1; 200.9)	0.08
	2	171.0 (144.7; 221.3)	154.9 (73.3; 174.9)	0.18
	4	161.0 (84.0; 231.4)	152.9 (143.3; 261.4)	0.97
	7	110.7 (113.7; 275.6)	110.2 (84.5; 196.2)	0.26
	1 year	184.7 (135.4; 267.0)	90.5 (4.0; 148.9)	0.02
IL-20, pg/mL, Me (Q25; Q75)	1	59.2 (46.8; 84.1)	48.9 (48.1; 60.8)	0.25
	2	63.5 (50.5; 90.2)	40.0 (28.8; 48.9)	0.005
	4	54.3 (45.0; 70.5)	43.5 (41.7; 51.1)	0.03
	7	57.9 (45.7; 73.3)	55.3 (38.9; 58.8)	0.34
	1 year	58.2 (43.7; 76.6)	56.9 (42.6; 69.6)	0.92
Oncostatin M, pg/mL, Me (Q25; Q75)	1	26.90 (7.44; 34.920)	21.48 (14.83; 25.36)	0.19
	2	25.82 (7.44; 34.49)	13.32 (9.12; 25.02)	0.16
	4	29.63 (9.41; 38.56)	16.56 (6.32; 23.83)	0.04
	7	38.62 (17.45; 53.20)	14.33 (6.91; 23.69)	0.002
	1 year	26.74 (17.71; 38.26)	12.33 (7.24; 20.90)	0.008
Placental GrowthFactor, pg/mL, Me (Q25; Q75)	1	10.96 (5.41; 23.39)	4.54 (0.34; 7.67)	0.02
	2	11.87 (5.34; 16.72)	3.15 (0.32; 8.73)	0.01
	4	8.07 (3.11; 16.86)	2.79 (0.84; 11.36)	0.12
	7	7.91 (5.20; 14.74)	2.56 (0.27; 3.95)	0.004
	1 year	7.73 (4.08; 12.27)	2.69 (1.35; 9.01)	0.04
sP-Selectin, pg/mL, Me (Q25; Q75)	1	69.45 (64.95; 79.81)	78.04 (47.25; 117.54)	0.04
	2	67.15 (61.15; 85.24)	86.79 (60.41; 120.79)	0.03
	4	68.87 (44.31; 83.77)	67.08 (44.81; 88.33)	0.85
	7	73.48 (49.98; 79.81)	66.74 (45.32; 87.43)	0.87
	1 year	82.63 (54.31; 92.93)	67.75 (55.71; 94.66)	0.77
LIGHT, pg/mL, Me (Q25; Q75)	1	195.76 (114.82; 245.30)	327.95 (194.89; 480.26)	0.13
	2	190.70 (167.49; 210.35)	274.00 (197.02; 443.96)	0.06
	4	199.45 (146.94; 305.82)	212.29 (110.12; 311.65)	0.82
	7	247.05 (182.33; 388.44)	263.53 (177.80; 322.51)	0.73
	1 year	226.17 (114.03; 392.93)	193.02 (83.02; 284.09)	0.53
Endocan-1, pg/mL, Me (Q25; Q75)	1	2467.50 (1542.0; 3489.0)	1861.0 (1369.50; 2630.50)	0.19
	2	1650.50 (1551.0; 3744.0)	1469.50 (1165.0; 3319.50)	0.13
	4	1240.50 (858.11; 1895.0)	1608.0 (1040.29; 2190.0)	0.16
	7	1287.00 (856.64; 1935.0)	1241.0 (1042.50; 1467.50)	0.78
	1 year	999.45 (786.86; 1171.0)	1005.49 (861.91; 1410.0)	0.26
sVCAM-1, pg/mL Me (Q25; Q75)	1	734.6 (700.04; 876.4)	612.09 (536.57; 739.7)	0.02
	2	696.6 (617.77; 744.1)	606.67 (543.68; 701.8)	0.08
	4	638.9 (569.93; 704.1)	593.60 (503.21; 653.1)	0.16
	7	674.6 (578.19; 714.5)	623.20 (446.58; 686.9)	0.16
	1 year	763.4 (668.08; 904.9)	600.47 (536.99; 643.0)	0.03

Note: XCL6—chemokine ligands 6; LIGHT—tumor necrosis factor ligand; CCL-15—leukotactin-1; CCL-21—6Ckine/Exodus-2; CCL-8—monocyte chemotactic protein-2; sVCAM-1—Serum Soluble Intercellular Adhesion Molecule-1.

**Table 4:** Dynamics of laboratory biomarkers in patients of the studied groups.

Indicator	Delta	MINOCA, n = 16	MI-CAD, n = 21	*p*-Value
CXCL6, pg/mL, Me (Q25; Q75)	1	−21.02 (−70.9; 15.74)	−10.50 (−56.1; 29.02)	0.59
	2	4.73 (−61.2; 45.25)	50.15 (−58.6; 163.07)	0.04
CCL-8, pg/mL, pg/mL Me (Q25; Q75)	1	3.72 (0.61; 7.65)	3.44 (−10.5; 13.6)	0.97
	2	−0.92 (−16.4; 14.7)	13.56 (2.28; 42.5)	0.04
CCL-15, pg/mL, Me (Q25; Q75)	1	−133.17 (−1999.0; 380.0)	−118.50 (−1969.0; −8.50)	0.39
	2	−154.0 (−598.0; 815.0)	−184.00 (−1177.0; 487.00)	0.93
CCL-21, pg/mL, Me (Q25; Q75)	1	76.6 (30.1; 126.3)	−91.75 (−145.4; −56.1)	0.03
	2	41.9 (−41.2; 124.6)	−6. 95 (−69.3; −45.2)	0.12
IL-20, pg/mL, Me (Q25; Q75)	1	0.45 (−5.5; 12.90)	1.0 (−13.7; 10.21)	0.38
	2	−1.75 (−7.0; 26.10)	3.85 (−5.2; 54.35)	0.42
Oncostatin M, pg/mL, Me (Q25; Q75)	1	−0.28 (−5.2; 8.23)	−4.26 (−15.1; 5.96)	0.13
	2	−2.54 (−14.9; 3.23)	−4.61 (−5.9; 11.02)	0.52
Placental Growth Factor, pg/mL, Me (Q25; Q75)	1	−1.19 (−8.1; 0.49)	0.29 (−1.3; 4.51)	0.17
	2	−1.84 (−2.5; 3.20)	0.63 (−0.1; 8.38)	0.11
sP-Selectin, pg/mL, Me (Q25; Q75)	1	10.61 (−1.1; 12.04)	−7.87 (−52.1; 8.88)	0.23
	2	7.02 (−4.0; 26.97)	−7.31 (−8.5; 25.85)	0.84
LIGHT, pg/mL, Me (Q25; Q75)	1	42.49 (−90.9; 178.78)	−82.92 (−363.9; −14.69)	0.03
	2	−73.68 (−101.4; 10.75)	25.38 (−89.9; 105.33)	0.72
Endocan-1, pg/mL, Me (Q25; Q75)	1	−1463.46 (−2423.0; −723.0)	−614.49 (−1526.5; −278.05)	0.03
	2	−246.50 (−691.0; −23.18)	−2.50 (−210.2; 830.40)	0.02
sVCAM-1, pg/mL Me (Q25; Q75)	1	34.96 (−12.7; 95.74)	−15.96 (−65.1; 53.16)	0.04
	2	131.43 (−31.9; 213.46)	−16.55 (−44.5; 156.38)	0.03

Note: Delta 1—the difference between the indicators after 1 year and 1 day; Delta 2—the difference between the indicators after 1 year and 7 days; CXCL6—chemokine ligands 6; CCL-15—leukotactin-1; CCL-21—6Ckine/Exodus-2; CCL-8—monocyte chemotactic protein-2; LIGHT—tumor necrosis factor ligand; sVCAM-1—Serum Soluble Intercellular Adhesion Molecule-1.

**Table 5:** Characteristics of the association of predictors with the probability of atherosclerosis progression in patients of the MINOCA group.

Predictors	Unadjusted		Adjusted	
	COR; 95% CI	*p*	AOR; 95% CI	*p*
sVCAM-1 (day 7)	0.996; 0.991–1.001	0.04 *	0.991; 0.984–0.998	0.02 *
CCL-21 (day 7)	1.004; 0.997–1.011	0.02 *	1.014; 1.002–1.026	0.02 *
LIGHT (day 1)	1.005; 0.998–1.010	0.07	1.008; 1.001–1.015	0.06
sP-Selectin (day 1)	1.004; 0.997–1.011	0.29	1.014; 1.002–1.026	0.08

Note: *—association of the outcome value with the predictor value is statistically significant (*p* < 0.05); CCL-21—6Ckine/Exodus-2; sVCAM-1—Serum Soluble Intercellular Adhesion Molecule-1; LIGHT—tumor necrosis factor ligand.

**Table 6:** Characteristics of the association of predictors with the probability of atherosclerosis progression in patients of the MI-CAD group.

Predictors	Unadjusted		Adjusted	
	COR; 95% CI	*p*	AOR; 95% CI	*p*
CCL-8 (day 7)	0.971; 0.944–0.999	0.04 *	0.941; 0.852–1.039	0.02 *
CXCL-6 (day 7)	1.016; 1.001–1.031	0.03 *	1.035; 0.977–1.095	0.02 *
LIGHT (day 7)	1.004; 0.997–1.011	0.19	1.018; 0.977–1.095	0.09
IL-20 (day 7)	1.000; 0.995–1.004	0.82	0.994; 0.987–1.001	0.12

Note: *—association of the outcome value with the predictor value is statistically significant (*p* < 0.05); CCL-8—monocyte chemotactic protein-2; CXCL6—chemokine ligands 6; LIGHT—tumor necrosis factor ligand.

## Data Availability

The datasets generated and analyzed during the current study are not publicly available, since they are in the process of patenting. The datasets used and/or analyzed during the current study are available from D. Vorobeva upon reasonable request.

## Supplementary Materials

The following supporting information can be downloaded at: https://www.mdpi.com/article/10.3390/jpm13121669/s1, Figure S1: indicators of multiplex analysis of blood serum of the studied groups; Figure S2: dynamics of laboratory biomarkers of the studied groups.
