# Supplementary material for: Pro-Inflammatory Biomarkers and Progression of Atherosclerosis in Patients with Myocardial Infarction with Non-Obstructive Coronary Artery Disease: 1-Year Follow-Up
Source: J Pers Med. 2023 Nov 29;13(12):1669. doi: 10.3390/jpm13121669 (PMC10744350; doi:10.3390/jpm13121669)
Supplement: Supplementary file 1 [file jpm-13-01669-s001.zip › graphics of the multiplex analysis_Supplementary.pptx]

## Slide 1
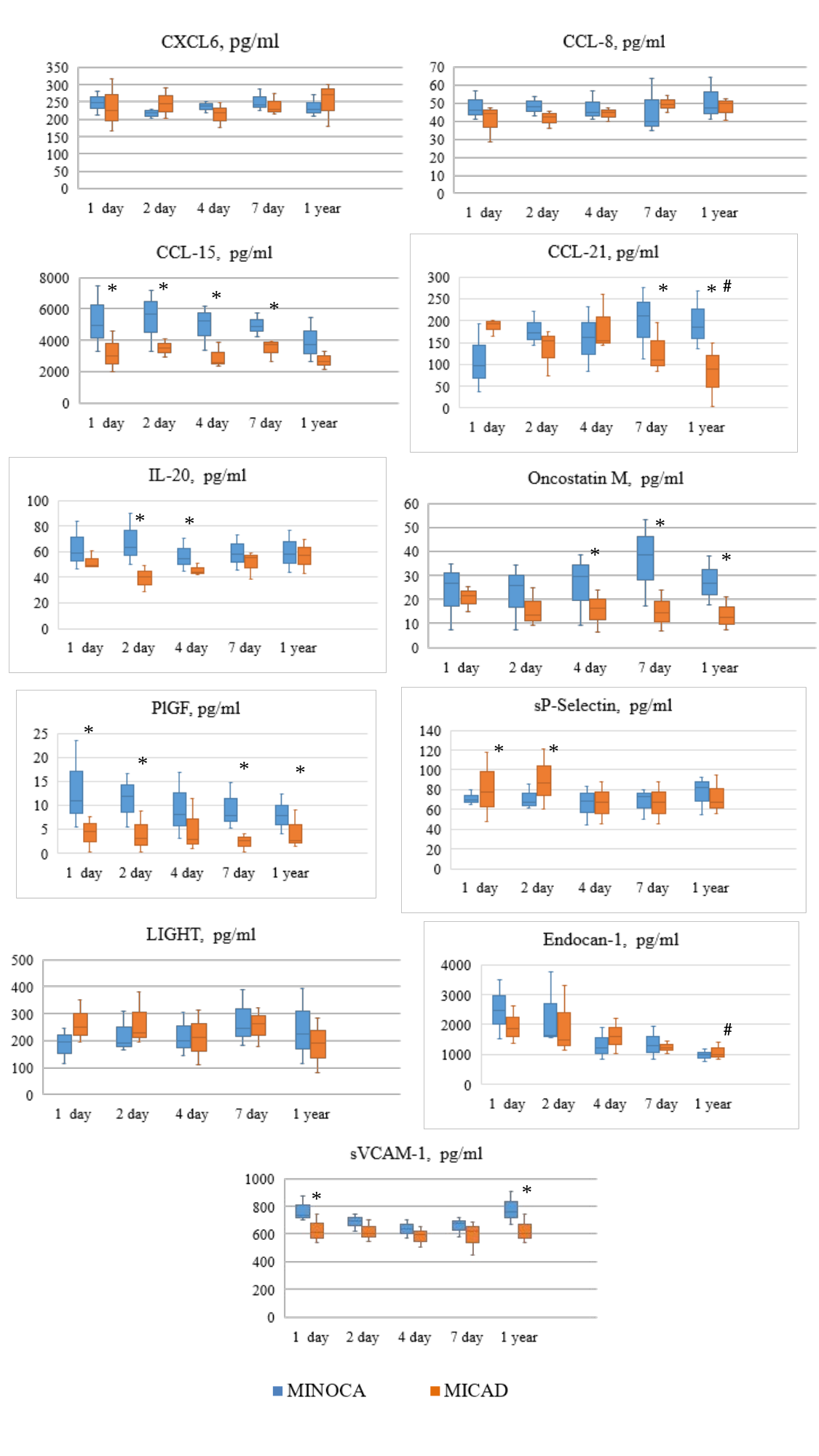

#
*
*
*
*
*
*
*
*
*
*
*
*
*
*
*
*
*
#
*
*

## Slide 2
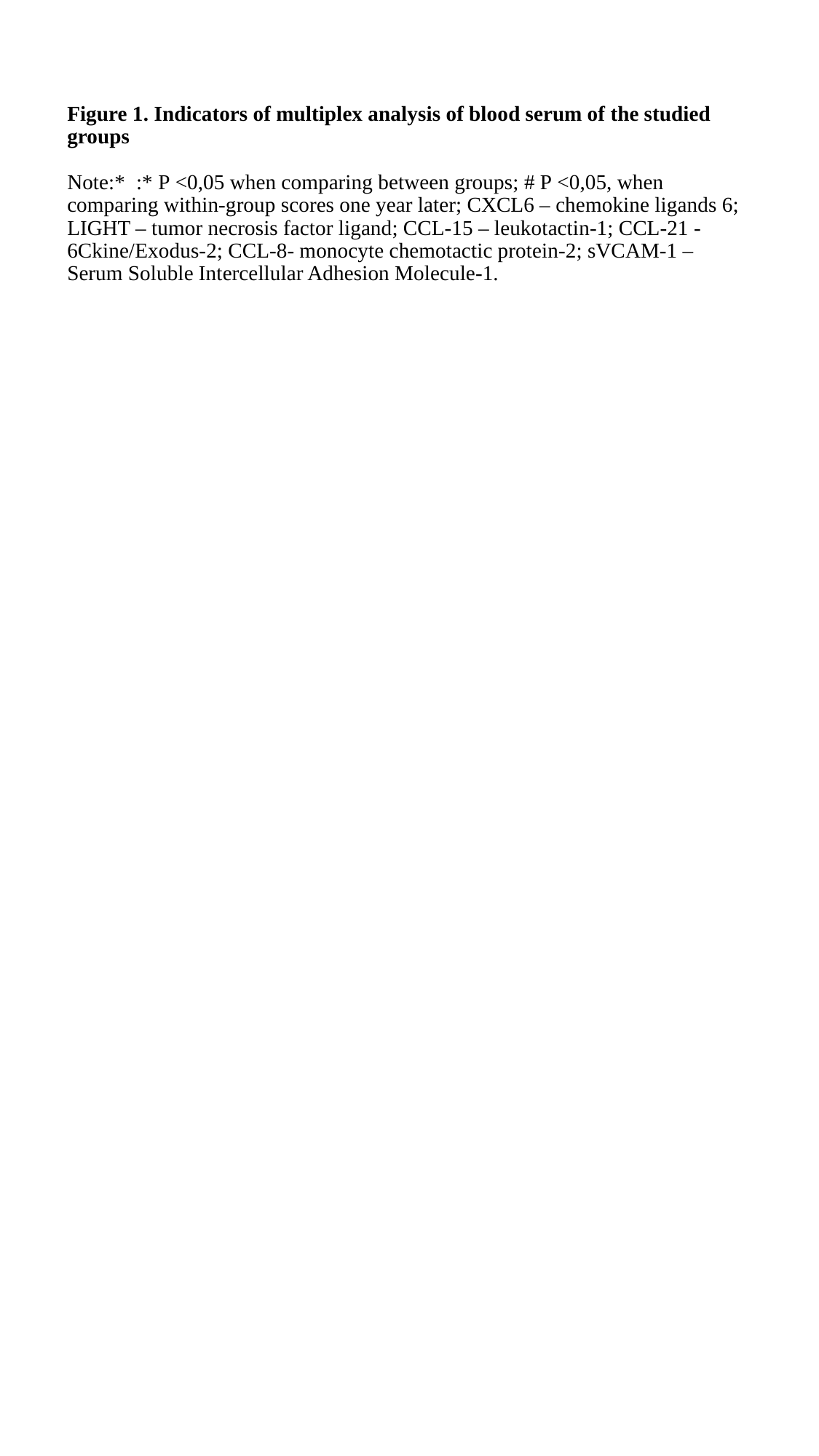

# Figure 1. Indicators of multiplex analysis of blood serum of the studied groups   Note:*  :* P <0,05 when comparing between groups; # P <0,05, when comparing within-group scores one year later; CXCL6 – chemokine ligands 6; LIGHT – tumor necrosis factor ligand; CCL-15 – leukotactin-1; CCL-21 - 6Ckine/Exodus-2; CCL-8- monocyte chemotactic protein-2; sVCAM-1 – Serum Soluble Intercellular Adhesion Molecule-1.

## Slide 3
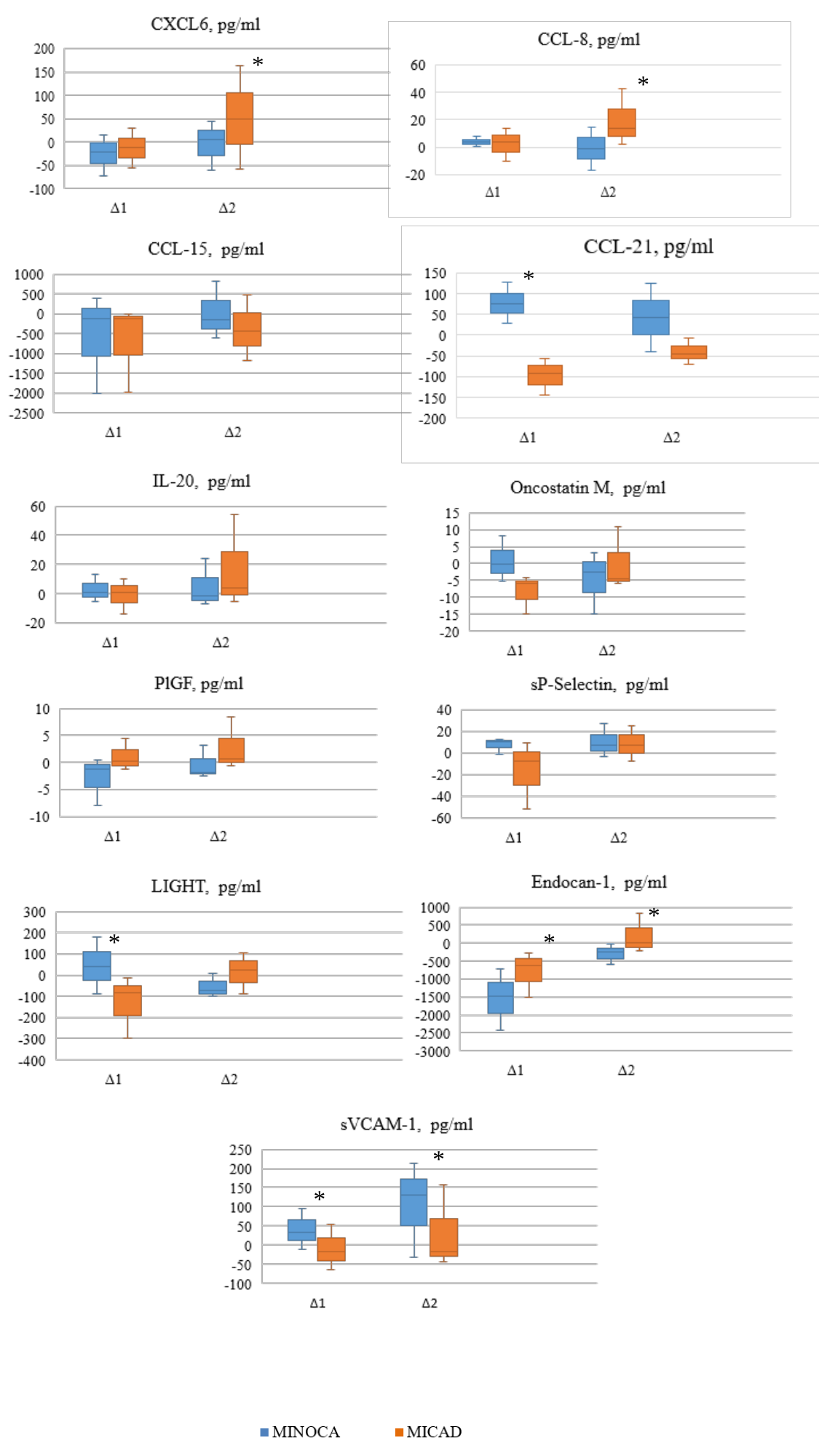

*
*
*
*
*
*
*
*

## Slide 4
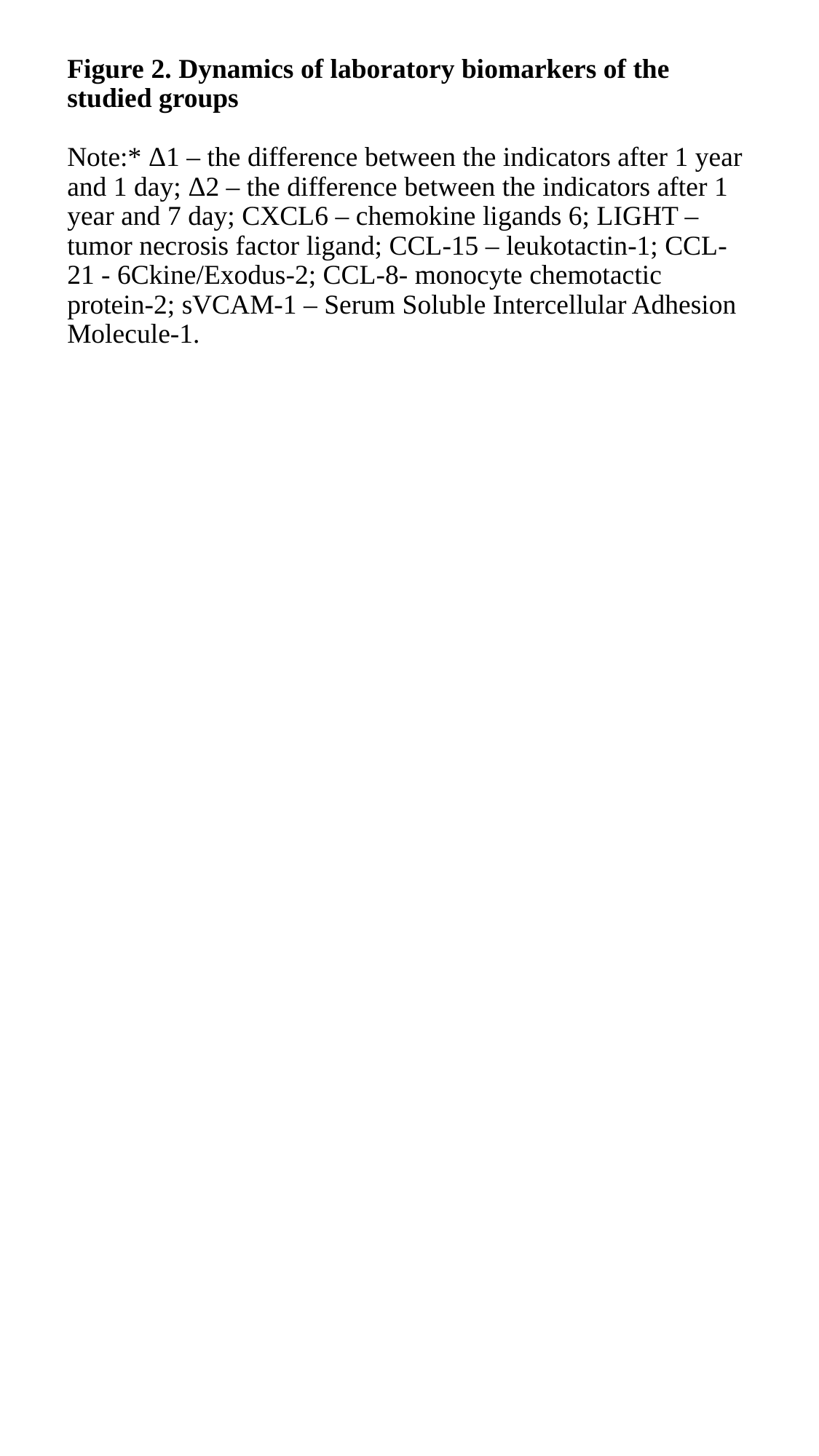

# Figure 2. Dynamics of laboratory biomarkers of the studied groups Note:* Δ1 – the difference between the indicators after 1 year and 1 day; Δ2 – the difference between the indicators after 1 year and 7 day; CXCL6 – chemokine ligands 6; LIGHT – tumor necrosis factor ligand; CCL-15 – leukotactin-1; CCL-21 - 6Ckine/Exodus-2; CCL-8- monocyte chemotactic protein-2; sVCAM-1 – Serum Soluble Intercellular Adhesion Molecule-1.
