# Supplementary figures and images for: Pro-Inflammatory Biomarkers and Progression of Atherosclerosis in Patients with Myocardial Infarction with Non-Obstructive Coronary Artery Disease: 1-Year Follow-Up
Source: J Pers Med. 2023 Nov 29;13(12):1669. doi: 10.3390/jpm13121669 (PMC10744350; doi:10.3390/jpm13121669)

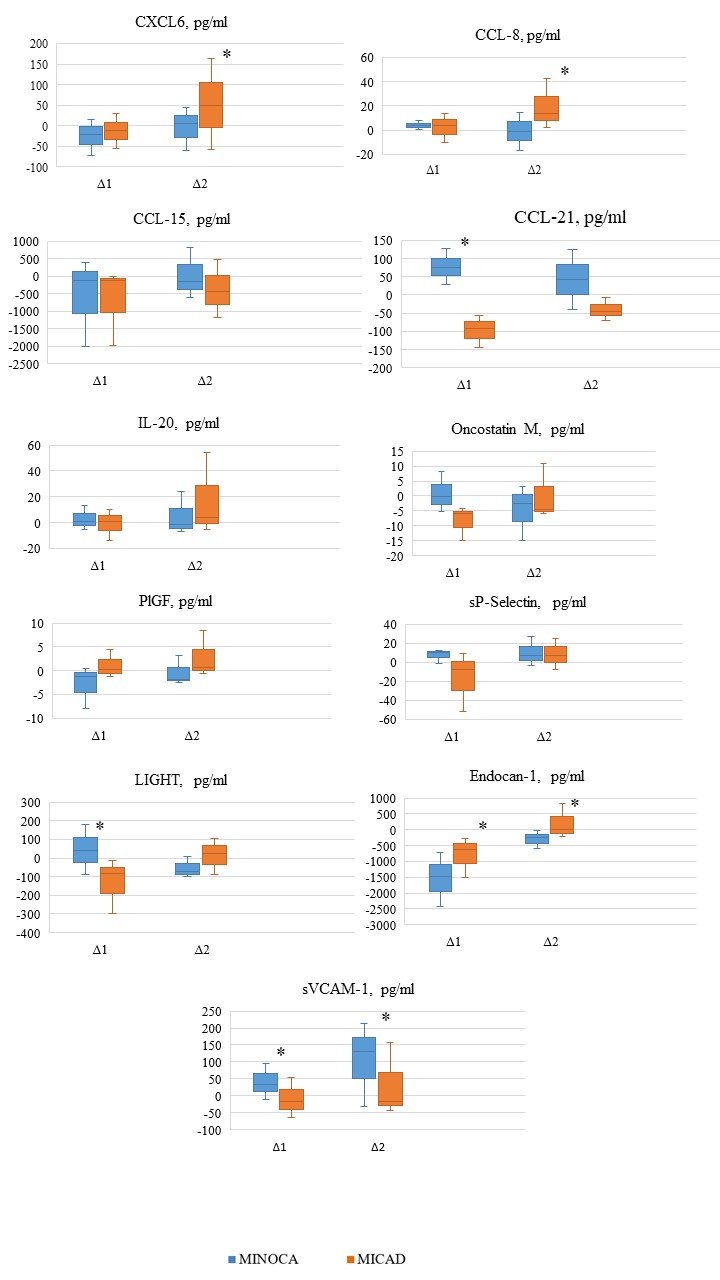

Supplement: Supplementary file 1 [file jpm-13-01669-s001.zip › Figura 2 - Dynamics of laboratory biomarkers of the studied groups.jpg]

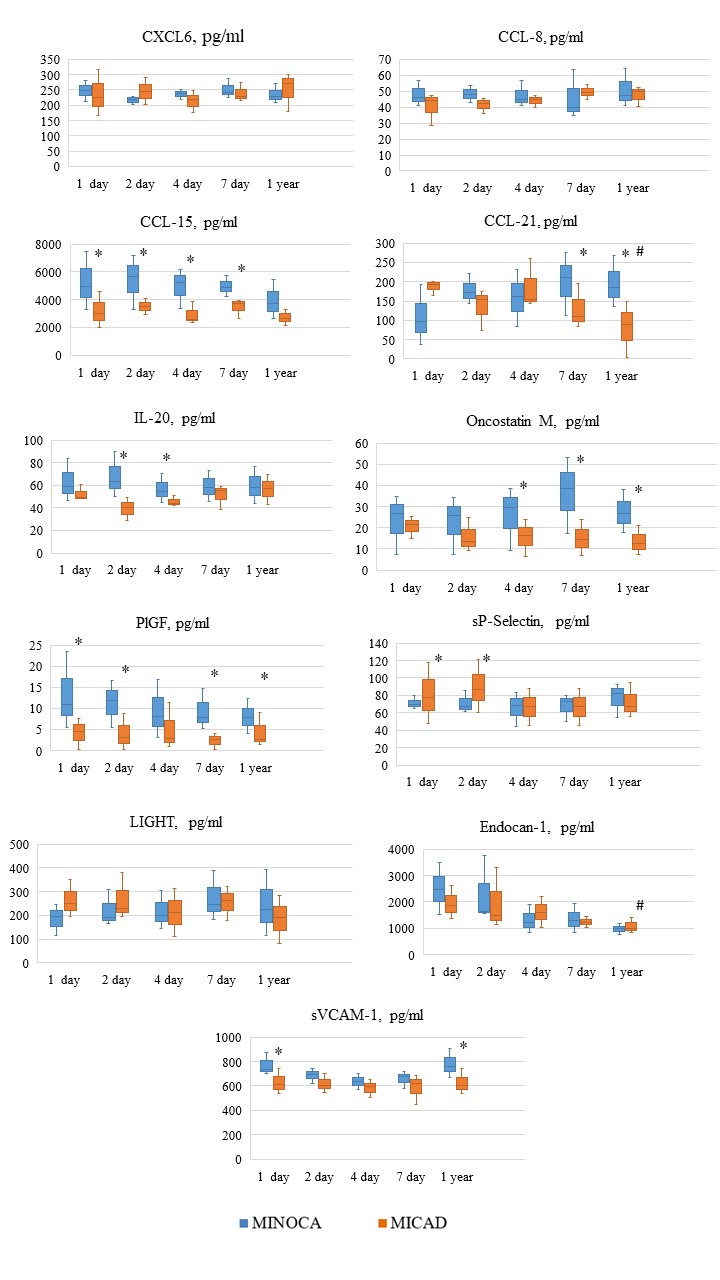

Supplement: Supplementary file 1 [file jpm-13-01669-s001.zip › Figura1 - Indicators of multiplex analysis of blood serum of the studied groups.jpg]
